# Malaria parasite carriage and risk determinants in a rural population: a malariometric survey in Rwanda

**DOI:** 10.1186/s12936-014-0534-x

**Published:** 2015-01-21

**Authors:** Fredrick Kateera, Petra F Mens, Emmanuel Hakizimana, Chantal M Ingabire, Liberata Muragijemariya, Parfait Karinda, Martin P Grobusch, Leon Mutesa, Michèle van Vugt

**Affiliations:** Centre of Tropical Medicine and Travel Medicine, Department of Infectious Diseases, Division of Internal Medicine, Academic Medical Centre, Meibergdreef 9, 1100 DE Amsterdam, The Netherlands; Medical Research Centre Division, Rwanda Biomedical Centre, PO Box 7162, Kigali, Rwanda; Royal Tropical Institute/Koninklijk Instituut voor de Tropen, KIT Biomedical Research, Meibergdreef 39, 1105 AZ Amsterdam, The Netherlands; Malaria & Other Parasitic Diseases Division, Rwanda Biomedical Centre, Kigali, Rwanda; Ruhuha Health Centre, Ruhuha Sector, Bugesera, Rwanda; College of Medicine and Health Sciences, University of Rwanda, Kigali, Rwanda

**Keywords:** Malaria, Prevalence, Risk factors, LLINs, IRS, Socio-economic status, Rwanda

## Abstract

**Background:**

Based on routine health facility case data, Rwanda has achieved a significant malaria burden reduction in the past ten years. However, community-based malaria parasitaemia burden and reasons for continued residual infections, despite a high coverage of control interventions, have yet to be characterized. Measurement of malaria parasitaemia rates and evaluation of associated risk factors among asymptomatic household members in a rural community in Rwanda were conducted.

**Methods:**

A malariometric household survey was conducted between June and November 2013, involving 12,965 persons living in 3,989 households located in 35 villages in a sector in eastern Rwanda. Screening for malaria parasite carriage and collection of demographic, socio-economic, house structural features, and prior fever management data, were performed. Logistic regression models with adjustment for within- and between-households clustering were used to assess malaria parasitaemia risk determinants.

**Results:**

Overall, malaria parasitaemia was found in 652 (5%) individuals, with 518 (13%) of households having at least one parasitaemic member. High malaria parasite carriage risk was associated with being male, child or adolescent (age group 4–15), reported history of fever and living in a household with multiple occupants. A malaria parasite carriage risk-protective effect was associated with living in households of, higher socio-economic status, where the head of household was educated and where the house floor or walls were made of cement/bricks rather than mud/earth/wood materials. Parasitaemia cases were found to significantly cluster in the Gikundamvura area that neighbours marshlands.

**Conclusion:**

Overall, Ruhuha Sector can be classified as hypo-endemic, albeit with a particular ‘cell of villages’ posing a higher risk for malaria parasitaemia than others. Efforts to further reduce transmission and eventually eliminate malaria locally should focus on investments in programmes that improve house structure features (that limit indoor malaria transmission), making insecticide-treated bed nets and indoor residual spraying implementation more effective.

## Background

Significant decline in malaria burden, attributed to scale-up of interventions including indoor residual spraying (IRS), insecticide-treated bed nets (predominantly long-lasting insecticide-treated net (LLIN) type) and use of artemisinin combination therapy (ACT) after confirmed diagnosis with microscopy or rapid diagnostic tests (RDTs), have been widely reported in multiple malaria-endemic countries, including Rwanda, during the last decade [1,2].

Following these gains, a new ‘Rwanda malaria control strategic plan 2013-2018’, aiming at achieving malaria pre-elimination status, with near-zero deaths from malaria and a slide positivity rate less than 5% among fever cases by 2018, is being finalized [3]. This change in strategy from successful individual case treatment (with a focus on reducing health facility-identified malaria cases) to improved large-scale control, reducing transmission (by increasingly targeting community-based, asymptomatic parasitaemic individuals and foci of infection) will require higher coverage and optimal use of implemented control measures and generation of area-specific, timely and accurate data to inform targeted control decisions [4]. For Rwanda, reported data stem from health facilities (HFs) that routinely monitor and report slide positivity rates (SPRs) that are important for surveillance [2,5]. These data are, however, representative of symptomatic cases captured by the health care system but not the total burden of malaria parasitaemic individuals, a significant proportion of whom are asymptomatic individuals in communities who are believed to be the reservoir pool for continued malaria transmission [6,7].

The epidemiology of asymptomatic malaria in the population (reservoir) is relevant information needed by control programmes to reduce both overall and area-specific malaria transmission, as well as to mitigate the effect of local malaria-transmission, foci-associated, risk factors. Currently, a major source of data on population level asymptomatic malaria parasitaemia is the nationally representative demographic and health surveys (DHSs) conducted every five years, which primary aim is to provide data for a wide range of monitoring and impact evaluation indicators in population, health, and nutrition issues [8]. However, because of the large coverage, DHSs are not powered to facilitate an accurate assessment of malaria reservoirs (asymptomatic-carrying, parasitaemic persons in a population in a given area) or to identify risk determinants of community-based, residual, malaria parasitaemia. The World Health Organization recommends field surveys that characterize baseline malaria transmission epidemiology with the aim of identifying *Plasmodium* spp. carriers and at-risk populations to inform targeted control for optimal impact [9]. Up to now, no study has been published on understanding malaria reservoirs and associated risk determinants in Rwanda.

As Rwanda embraces a transition towards achieving malaria pre-elimination status, it becomes very important to know the specific local determinants that predict parasite carriage. This paper describes a community-based, malariometric survey to measure baseline parasite carriage rates and to study associated risk factors of residual malaria parasitaemia in order to optimize malaria control interventions targeted to specific local needs.

## Methods

### Study site and population

**Figure 1:**
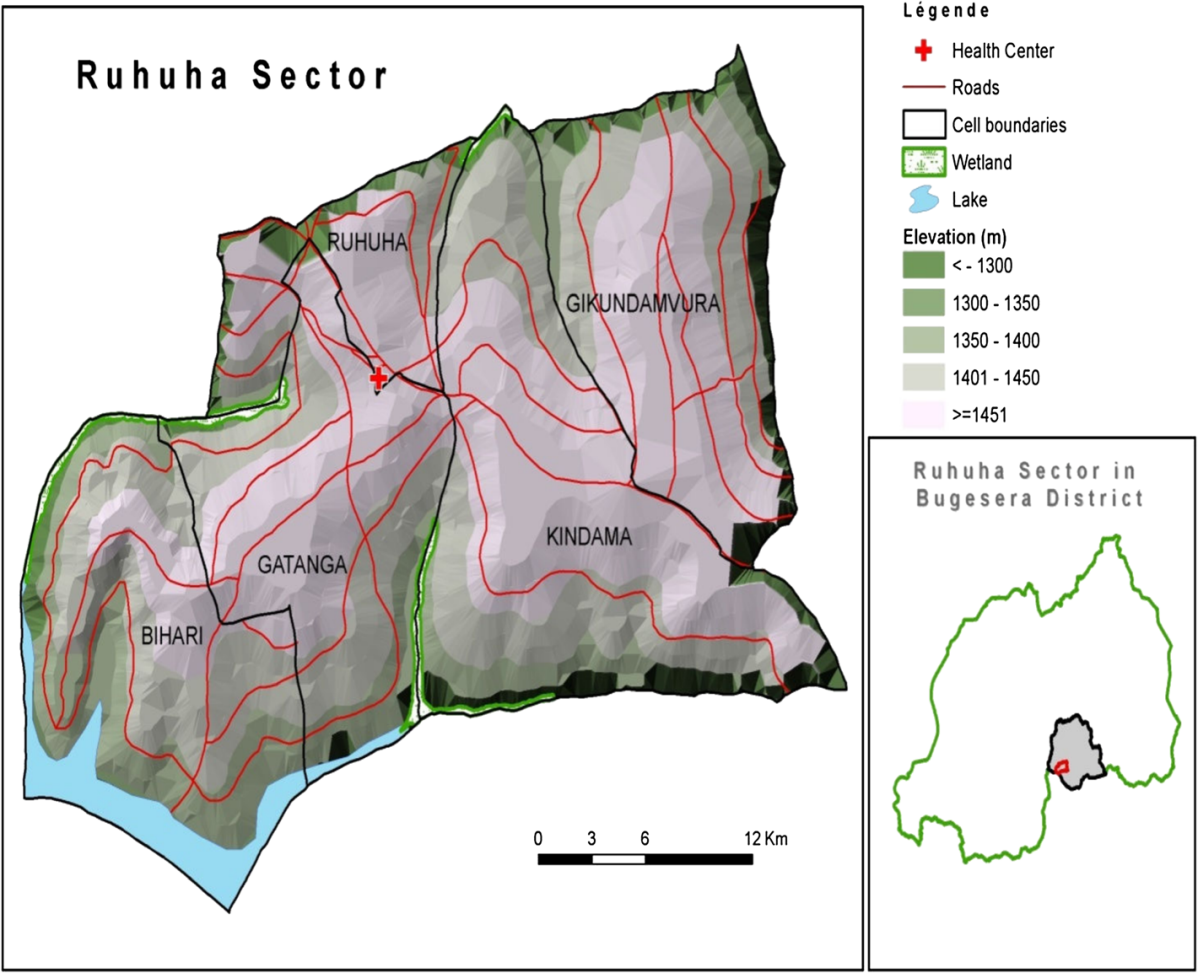
**Map showing five cells that constitute Ruhuha Sector and the sector (red circle) location in Bugesera District (grey polygon) in Eastern Province, Rwanda.**

Geopolitically, Rwanda is divided into provinces, districts, sectors, cells, and villages with district being the basic political administrative unit. This study was conducted in 35 villages located in five cells that constitute Ruhuha Sector (Figure 1), a rural, agricultural, traditionally high malaria transmission setting in eastern Rwanda. The area experiences two high malaria transmission peaks associated with rainy seasons observed generally from October to November and March to May. The reported total sector population was 21,606 individuals living in 5,100 households (Ananie Sibomana, pers. comm.). Study eligibility criteria included: 1) having spent the night prior to the interview in a studied household (HH); 2) aged ≥ six months; and, 3) provision of informed consent.

### Study design and selection of study participants

To provide baseline assessment of local malaria transmission and informed decision-making on follow-up interventions, a sector-wide, HH-based, cross-sectional survey was conducted between June and November 2013 (rainy season was late August to November). In summary, the night prior to the survey, a designated village area community health care worker (CHW) identified HHs to be visited from an enumeration list and proceeded to request the head of household (HoH) (a self-reported principal responsible adult ≥18 years) and HH members to stay at home at the appointed survey date if possible. The survey consisted of two parts: a questionnaire administered to the HoH and a laboratory survey in which all HH members were asked to participate. On the survey day, the study team members, including a laboratory technician and an interviewer (in company of the CHW) visited the prior-notified HH and proceeded to administer the questionnaire and perform all study clinical evaluations (see Laboratory methods) after the HoH had provided written consent. Where no member was found present in an HH, a return visit was scheduled in the next seven days to optimize study enrolment; in case the survey was not conducted on this follow-up visit, the HH was omitted from study enrolment.

### Questionnaire and interviews

An interviewer-administered questionnaire was held with the HoH. Information on demographics (sex, age, literacy, occupation, religion, and marital status); malaria prevention measures ((LLIN ownership, (number and use, and IRS history); HH structural features (type of wall, floor and roof); prior fever management practices and socio-economic status indicators (HH utilities like water source for domestic use, lighting and cooking) was collected. The questionnaire, written in English language, was field-tested at three sites to ensure consistency and comprehension. Field workers were trained, across all subject areas and related questions, to administer the interviews in the local dialect (*Kinyarwanda*). Questionnaire data were collected in electronic form using Open Data Kit (ODK) Collect setup [10]. ODK is an open-source suite of tools that include ODK Collect, an android-based mobile client that acts as the interface between the user and the underlying form used to collect data [10]. The collected data were then electronically loaded onto a central server.

### Laboratory methods

Study participants were asked to provide a finger-prick blood sample for malaria diagnosis. A thick blood smear was prepared, dried and stained with 2% Giemsa immediately in the field and later. Light microscopy was performed at Ruhuha Health Centre (RHC). Two trained technicians independently examined all blood smears and a third reader was used in the event of any discordant readings between the two readers. Experienced microscopists at the National Reference Laboratory in Kigali performed quality control for all positive slides and 5% of all negative smears. Asexual stage parasites were counted per 200 white blood cells (WBC). A blood smear was considered positive in the presence of any asexual parasites and negative if examination of 100 high-power fields did not reveal any asexual parasites. Field laboratory data were collected and transcribed directly into hard-copy field laboratory registers and later entered into Microsoft Access software.

### Statistical analysis

Laboratory and questionnaire data were merged and entered into STATA version 12.1 (STATA Corp., College Station, TX, USA) for analysis. Data analysis was conducted in two parts: at HH and individual level to ensure adjusting for within- and between-HH correlations. Univariate logistic regression was used to assess the effect of predictor variables on the primary outcome. All variables with possible malaria risk association (p <0.15) were included in subsequent adjusted multivariate logistic regression models. At individual level, a random effects model was used to adjust for within- and between-HH clustering, allowing for a reduced weighting for each subsequent malaria-parasitaemic individual recorded from a HH after the index cases. At HH level, a stepwise backwards-elimination approach was used in the multivariate logistics regression model to exclude any variable with no significant effect. At both levels, malaria risk statistical significance was considered for any variable with an effect associated with a p-value >0.05. Wald tests were used to analyse the effect of included variable in the model on the primary outcome.

The dependent variables for this study were: 1) malaria parasitaemia per individual, defined as the presence of any asexual parasites in the blood smear examined by light microscopy; and, 2) malaria parasitaemia per HH, defined as the presence of asexual malaria parasites detected on a thick peripheral blood smear for at least one HH member. Independent study variables included individual and HH demographic data (age, sex, religion, marital status, area of residence), socio-economic indicator variables (see section below), reported knowledge on malaria prevention practices (including availability and use of LLINs, HH use of IRS as well as reported prior fever management experiences), and household structural features, including type of roof, floor and wall material.

### Household socio-economic status (SES)

In total, nine SES indicator variables (Table 1) were used to generate a SES score for each HH by principal component analysis (PCA) as described elsewhere [11]. The PCA output was taken as a weight for each variable and the sum of the weights for each HH taken as the dependent variable household’s SES score. The scores were then ranked in terciles with the highest 33% of HHs considered high SES, the lowest 33% as low SES and the rest as middle SES [12].

**Table 1:** **Baseline demographic, household and malaria control characteristics**

**Demographics**	**n**	**%**	**Household SES indicator variables**	**n**	**%**
**HoH level of education**			Does HoH belong to an economic group?		
None	1,414	(35.63)	No	1,807	45.53
Primary	2,056	51.80	Yes	2,162	54.47
Secondary	374	9.42	Does HoH have health insurance?		
Tertiary	125	3.15	No	1,337	33.69
**HoH religion**			Yes	2,632	66.31
Catholic	1,440	36.28	Has HH saved any money in last 3 months?		
Protestant	1,330	33.51	No	3,163	79.7
Moslem	72	1.81	Yes	806	20.3
SDA	806	20.32	Does HH own current house of residence?		
JHW	43	1.08	No	698	17.6
No religion	251	6.32	Yes	3,271	82.4
Others	27	0.68	Source of water for domestic use		
**HoH marital status**			Open (well, lake)	1,624	40.9
Never married	444	11.89	Closed (piped water)	2,345	59.1
Married	1,663	41.89	Type of material house wall is made of		
Living together	886	22.32	Mud/wood	1,171	29.5
Separate/Divorced	255	6.42	Cement/bricks	2,798	70.5
Widow/widower	717	18.08	Type of material house floor is made of		
**HoH main occupation**			Earth/clay/dung	3,136	79
Farmer	3,073	77.43	Bricks/cement	833	21
Public office	171	4.31	HH source of power for cooking		
Self employed	326	8.21	Firewood/straw	3,787	95.4
Private officer	170	4.28	Electricity/charcoal	182	4.6
Student	31	0.78	HH source of power for lighting		
Unemployed	92	2.32	Kerosene/candles/firewood/touches	3,385	85.3
Others	106	2.67	Electricity	584	14.7
**HH wealth and occupancy**					
Any birth in HH in last 5 years?			**Malaria control variables**		
No	1,792	45.15	HH bed net ownership of at least 1 net		
Yes	2,177	54.85	No	282	7.11
Number of persons in HH			Yes	3,687	92.89
1-3	1,493	37.62	IRS done in last 6 months?		
4-5	1,454	36.63	No	217	5.47
6-7	757	19.07	Yes	3,752	94.53
8+	265	6.68			
SES score					
Low	1150	33.4	Household with at least 1 case of malaria	518	13.05
Medium	1147	33.3	Household without any malaria carriers	3,450	86.95
High	1146	33.3			

### Study consent and ethical approval

Written informed consent was obtained from the HoH and for all HH members aged ≥12 years. Verbal consent was obtained for blood slide preparation. Study protocols received ethical and scientific approved by the National Health Research Committee (NHRC) and the Rwanda National Ethics Committee (No 384/RNEC/2012), Kigali, Rwanda.

## Results

### Study population

**Figure 2:**
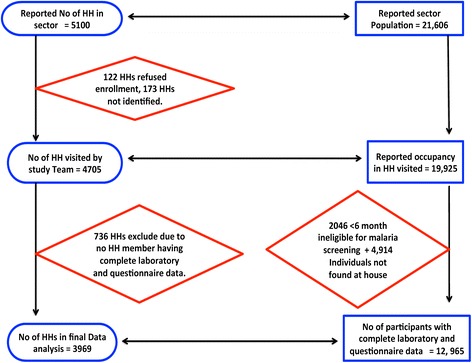
**Flow chart of study household/participant enrolment and malaria screening.**

In total, 4705 households occupied by 19,925 individuals were surveyed. In the final analysis, only data from 12,965 (65%) eligible individuals (3,968 households), who had complete questionnaire and laboratory data on all covariates, were included. A flow chart of the survey process and selection of participants is detailed in Figure 2. A greater proportion of study participants were female (53.5%) and the age distribution was 15.1, 32.58 and 52.31% for age groups six to 59 months, five to 15 years and ≥16 years, respectively (Table 2).

**Table 2:** **Univariate and multivariate regression analysis of individual risk factors for malaria slide positivity**

**Variable**	**N =** **12,965** **n (%)**	**Univariate analysis**	**Multivariate analysis**
		**OR (95% CI), P value**	**OR (95% CI), P value**
**Malaria infection** (positive)	652 (5.03)	--	--
**Gender**			
Female	7,567 (58.36)	1	1
Male	5,398 (41.64)	1.409 (1.191-1.667), <0.0001	1.201 (1.009-1.428), 0.039
**Age group**			
0-4	2,199 (16.96)	1	
5-15	4,431 (34.18)	1.905 (1.514-2.397), <0.0001	1.938 (1.541-2.438), <0.0001
16+	6,335 (48.86)	0.359 (0.275-0.468), <0.0001	0.384 (0.294-0.503), <0.0001
**Fever in last 6 months**			
No	4,838 (37.32)	1	1
Yes	8,127 (62.68)	1.464 (1.209-1.773), <0.0001	1.306 (1.072-1.590), 0.008
**HH wall types**			
Bricks/cement	3,780 (29.16)	1	11
Wood/mud	9,185 (70.84)	0.550 (0.458-0.661), 0.001	0.543 (0.442-0.668), <0.0001
**HH roof type**			
Wooden poles	24 (0.19)	1	1
Tiles/Iron sheets	12,941 (99.81)	0.558 (0.037-0.933, 0.04	0.239 (0.053-1.074), 0.062
**HH floor type**			
Clay/Earth/Dung	10,301 (79.45)	1	1
Cement/bricks	2,664 (20.55)	0.384 (0.289-0.511), <0.0001	0.529 (0.389-0.719), <0.0001
**Wealth index**			
Low	3,608 (27.83)	1	
Medium	6,672 (51.46)	0.618 (0.509-0.751), <0.0001	0.726 (0.592-0.890), 0.005
High	2,685 (20.71)	0.479 (0.3760-0.610), <0.0001	0.599 (0.451-0.797), 0.002
**Residential cell**			
Biharwe	2,249 (17.35)	1	
Gatanga	2,822 (21.77)	0.920 (0.678-1.250), 0.595	1.016 (0.741-1.392), 0.923
Gikundamvura	2,565 (19.78)	1.883 (1.418-2.504), <0.0001	2.432 (1.797-3.293), <0.0001
Kindama	3,341 (25.77)	1.023 (0.977-1.304), 0.876	1.487 (1.091-2.025), 0.012
Ruhuha	1,988 (15.33)	0.631 (0.440-0.905), 0.012	0.957 (0.650-1.408), 0.822
**Malaria control tools used**			
**IRS done in HH**			
No	217 (5.47)	1	
Yes	3,752 (94.53)	1.150 (0.729-1.815), 0.549	
**Own ≥1 LLIN in HH**			
No	282 (7.11)	1	
Yes	3,687 (92.89)	1.144 (0.761-1.722), 0.517	

### Malaria prevalence, control intervention coverage and fever management

Overall, individual *Plasmodium* parasite carriage prevalence was 5.03% (95% CI 4.65-5.41%). At HH level, 518 HHs (prevalence of 13% (95% CI 12.01-14.10%) had at least one member with malaria parasitaemia. HH ownership of ≥ one LLIN was 92.9% (95% CI 92.193.7%) and the proportion of HHs where IRS had been conducted within 12 months prior to survey was 94.5% (95% CI 93.8-95.2%). In 2,254 (56.8%) HHs, at least one member was reported to have had fever in the previous six months and in 1,277 (32.2%) of these HHs, fever was reported to have occurred in the four weeks prior to the survey date. Of the reported fever cases, 1,654 (41.67%) were treated in the government health care system, 449 (11.31%) purchased drugs from the pharmacy, while 151 (3.8%) used either local medicinal herbs or home-based, malaria medications from previous episodes.

### Univariate analysis

#### Individual risk factor analysis

Results of the univariate analysis (with adjustment for within- and between-household clustering) are shown in Table 2. Sex (males had 1.4-fold increase in odds), age groups (with age-groups five to 15 years and ≥16 having 1.9 and about 0.4 times more risk than children aged six to 59 months, respectively) and a reported history of fever during the previous six months (1.46-fold higher odds of parasitaemia) showed a significant risk effect. Significantly higher malaria risk was also associated with SES-related variables. House structural features had significant effect on malaria risk. Living in houses with cement/brick walls had a reduced risk (odds ratio: 0.55) odds of parasitaemia compared to wood/mud-walled houses. Living in houses roofed with tiles/iron sheets versus straw/wooden planks/tent roofs was associated with a reduced risk (odds ratio: 0.56) of parasitaemia and living in houses with cement/bricks floors versus clay/mud/dung floors was associated with a reduced (odds ratio: 0.38) risk of parasitaemia.

### Household risk factor analysis

Results of the univariate analysis for HH level risk determinants are shown in Table 3. In summary, the risk of finding parasitaemia at HH was significantly higher with increasing number of HH occupants. However, the risks were lower in HHs where the HoH had any level of education (OR = 0.777 (95% CI 0.634-0.952), was able to save some money in the previous three months (OR = 0.675, 95% CI 0.524-0.869), had any form of health insurance (OR = 0.759 (95% CI 0.628-0.919), and where the HH had parameter values associated with a medium and high SES class.

**Table 3:** **Baseline household characteristics, univariate and multivariate analysis**

	**Univariate analysis**	**Multivariate analysis**
	**OR (95% CI), P value**	**OR (95% CI), P value**
**Household demographics**		
**HoH education level**		
None	1	
Primary - Tertiary	0.777 (0.634-0.952), 0.015	0.810 (0.655-0.999), 0.05
**Occupation**		
Farmer	1	
Public office	0.829 (0.425-1.617), 0.582	1.287 (0.634-2.609), 0.485
Self employed	0.581 (0.381-0.888), 0.012	0.789 (0.506-1.231), 0.297
Private officer	0.467 (0.168-1.296), 0.144	0.667 (0.233-1.908), 0.45
Student	1.890 (0.754-4.737), 0.174	3.076 (1.121-8.436), 0.029
Unemployed	0.756 (0.376-1.522), 0.434	0.85 (0.411-1.756), 0.66
Others	0.713 (0.305-1.671), 0.437	0.740 (0.309-1.774), 0.5
**Number of persons in HH**		
1-3	1	1
4-5	2.555 (1.976-3.303), <0.0001	2.504 (1.895-3.309), <0.0001
6 +	4.102 (3.167-5.314), <0.0001	4.911 (3.702-6.517), <0.0001
**Household structure features**		
**Type of house wall material**		
Mud/wood	1	1
Cement/bricks	0.622 (0.513-0.753), <0.0001	0.706 (0.567-0.878), 0.002
**Type of house floor material**		
Earth/clay/dung	1	1
Bricks/cement	0.381 (0.283-0.513), <0.0001	0.640 (0.435-0.941), 0.023
**HH source of power for lighting**		
Kerosene/firewood/touches	1	1
Electricity	0.194 (0.122-0.310), <0.0001	0.258 (0.142-0.466), <0.0001

### Multivariate analysis

#### Individual level predictors

In the multivariate analysis (Table 2), significant malaria parasitaemia risk factors that remained were sex (male associated with a OR = 1.2), age group (with five to 15 year olds having a 1.94-fold increase while individuals of age group ≥16 year had a reduced risk (OR = 0.38)), a reported history of fever and study participant residential cell. As in HH level predictors, parameters HH floor, roof and wall material types, values associated with medium and high SES levels, were associated with significantly lower odds of parasitaemia (Table 2).

### Household level predictors

In the multivariate model (Table 3), significant HH level malaria risk effect was associated with HoH reported education level, occupation, housing structural features (walls and floors that were constructed with cement/brick had a protective effect of OR = 0.706 (p = 0.002) and OR = 0.640 (p = 0.023), respectively), source of lighting (electricity was associated with reduced (OR = 0.258, p = <0.0001)). Malaria risk also varied by number of people living in a HH.

## Discussion

In this study, malaria parasite carriage prevalence was 5.03% among study participants, and 13% of HHs had at least one malaria-parasitaemic member. Risk factor analysis identified variables that, alone or in combination, significantly influenced risk of malaria to include age group, sex, administrative cell of residence, number of HH occupants, HH structural features, and HH SES indicators. LLIN ownership and IRS activity were not associated with malaria risk.

Malaria parasite carriage prevalence among all age participants was 5 and 9.7% among children two to ten years. In an earlier study in this area, asymptomatic parasitaemia rates among HH members (of fever cases identified at the hospital) was 5.1%, suggesting that asymptomatic carriage rates have remained stable over the last two years [7]. Parasite carriage rates in a community are a marker of malaria endemicity since they correlate with the frequency and duration of parasite exposure [13]. Based on endemicity classifications, the area studied was at hypo-endemic transmission level (<10% parasite rates in children two to ten years).

However, some areas within the Ruhuha sector showed significantly higher malaria transmission. Living in Gikundamvura cell was associated with a significantly increased malaria risk, relative to the other residential cells. A similar finding was also shown in 2011 [7]. Gikundamvura is an area surrounded in the northeast by a vast expanse of marshland used for rice cultivation, which is a major source of food and income. It is plausible that the marshlands support mosquito breeding and increased malaria transmission risk for neighbouring HHs. A follow-up study on environmental, entomological and spatial risk features to better characterize the observed high malaria risk is planned.

The studied area showed a high IRS coverage and LLIN ownership (both over 90%). However, neither LLINs nor IRS showed any significant effect on malaria risk in this area. With respect to LLINs, possible reasons for no observed protective effect may include infrequent net use and poor quality of nets being used poor quality of nets being used *as reported elsewhere* [14]. In a previous study in this area, only in 18% of visited HHs was a bed net found to be physically hung onto a bed or sleeping space suggesting that bed net use may be sub-optimal and that ownership of a bed net does not automatically lead to usage of the net [7]. It is also plausible that most malaria-causing bites occur in the evening and early night hours when most individuals are still outdoors and use no control measure. Additionally, a change in mosquito biting preferences to biting outdoors may increase risk of *Plasmodium* parasite transmission despite the population having and using recommended malaria prevention indoor control measures.

Males were associated with higher malaria risk in this study, as has been shown in comparable settings elsewhere, suggesting that males may exhibit a behaviour pattern subjecting them to higher risk of exposure [15]. However, other studies, including one previous study from this area, have shown either no sex differences in malaria risk, or with the risk changing across sex by seasonality [7,16-18]. Either inherent differences or social, occupational or cultural determinants of exposure risk behaviour across different settings may explain these observed risk difference by sex.

Age is an established risk factor for malaria, although its effect is influenced by area-specific endemicity levels [15,19,20]. In this region, reported routine data (slide positive rates) suggested reduced malaria transmission after the scaling-up of LLINs and IRS coverage in 2000–2010 [2]. This transition in malaria transmission may have influenced age-related risk of malaria parasitaemia. Compared to children under four years, children aged five to 15 years, had increased odds of malaria risk while individuals aged ≥16 years had significantly lower risk of parasitaemia. Other studies in Kenya and Eritrea demonstrated an increased higher risk in older age groups relative to < five year olds in numeric order [21,22,15]. Similarly, a prior study conducted in Ruhuha [7] showed a significantly higher risk in older age groups. In particular, a shift in the age at which malaria peak prevalence was observed towards older children has been seen where mosquito net coverage has increased concomitantly [20], and in association with reducing entomological inoculation rates (EIRs) [23]. A reduction in exposure to *Plasmodium* spp. inoculation leading to delays (in older age groups) or failure in acquiring protective immunity is unlikely to account for the lower risk in the older age groups as they were carrying asymptomatic parasitaemia and hence had not lost their immunity to malaria.

Human activity and mosquito-biting habits may also play a part in differential mosquito-human exposure patterns. Behavioural patterns, including older children working and playing where the *Anopheles* vector is present, especially at dusk when *Anopheles* becomes active, have been suggested elsewhere [24]. Apart from younger children being more likely to sleep under bed nets compared to older siblings [25,15], older children, as observed in this area, stay out longer in the evening and are more likely to be bitten by malaria-carrying mosquitoes outdoors before returning later to their households. In the Nigeria Garki malaria elimination project a major reason for failure to achieve elimination was poor control of transmission, important outdoor-feeding and resting vector populations [26]. Age-group differences in risk of exposure to mosquito bites including use of malaria preventive measures like LLINs are more plausible reasons for the observed risk of parasitaemia patterns in this study.

In this study, an increasing malaria risk was associated with higher house occupancy. In a recent study in southeastern Tanzania, mosquitoes were found to be more attracted to houses with high occupancy [27]. The presence of multiple sleepers leads to production of larger volumes of mosquito-attracting human emanations and hence the increased risk of transmission in comparison to houses with lower occupancy [28,29].

House structural features, such as types of floor, roof and wall material, have previously been shown to influence risk of malaria infection [16,22,30,31]. Study findings confirmed that HH features associated with ease of entry, hiding and resting places within HHs, factors that favour mosquito survival, biting and transmission chances, pose a higher risk of malaria parasitaemia. HHs with wall structures made of bricks and cement (*vs* wood and mud) and whose floor was made of bricks/cement (*vs* earth/dung/clay) had a protective effect. Houses made of poor quality wall and roof materials are likely to have eaves and openings that allow mosquitoes to easily access and stay longer in HH [32]. In this study, type of roofing was not a significant risk determinant, but this could be because 99.3% of all houses in the area are roofed with iron sheets and not enough statistical power could be generated to see an effect. This study highlights the potential value of improved house design to prevent mosquito entry and to minimize risk of indoor malaria transmission as efforts supplementary to maintaining high coverage of other interventions, including IRS and LLIN [27].

Compared to low SES HHs, medium and high SES HHs were associated with 0.73 and 0.48-fold reduction in risk of parasitaemia. Similarly, a malaria parasitaemia protective effect found in HHs of high SES has been previously reported [33-36]. In one study, improving house structural features was associated with lower malaria risk, possibly due to better restriction of mosquito entry [37]. These findings are particularly consistent with studies based on confirmatory parasitaemia as opposed to self-reported malaria/fever classifications [33,38,39]. Other socio-economic indicator variables associated with a reduced malaria risk for family members included HHs, where HoH reported having an education (*vs* no education) and where the HH main source of lighting was electricity (*vs* kerosene/candles/firewood/torches). Both variables are a proxy measure of higher SES, a feature associated with lower risk. A possible reason for this may be that high SES individuals may have a higher purchasing capacity for, and access to malaria-protective measures including better housing facilities. Conversely, HHs where the HoH reported to be a student (as the principal occupation) were associated with a higher risk of having a HH with malaria.

This study has several limitations. To ensure all HH in the study area were visited, enumeration lists generated by CHWs were used. However, during study implementation, a number of HHs could not be found and there was no systematic strategy to identify these missing households. Another limitation may be the detection method of malaria. Malaria parasitaemia was diagnosed by light microscopy, which is known to have a lower detection limit compared to molecular methods, especially in cases with low parasitaemia. This may have underestimated the malaria burden, especially for asymptomatic cases that tend to have low parasite carriage rates. In addition, the survey period covered (June to November) was longer than initially planned (June to August). This period covered times when both primary and secondary schools were either open or closed (during school breaks) as well as before and after rainy season periods. For households visited during the school season, many of the schoolchildren were not present in the HH, and laboratory data could not be captured and were hence missed in the final analysis, which may have limited study representativeness. Because reported study results were derived from a cross-sectional survey, associations observed may be confounded by unmeasured factors and are not suitable for drawing causal inferences. Areas visited during the rainy season may have had a greater risk of malaria than those visited outside the rainy period (such as Gikundamvura). However, in a previous study done in the same sector [7] that had no seasonality bias, Gikundamvura cell showed a greater risk as well, indicating that the rainy season may not have significantly influenced malaria parasitaemia risk in this area.

## Conclusion

Study results demonstrated malaria-hypoendemic levels of transmission, with the distribution shown to vary spatially in this area. Age, sex, house structural features, and socio-economic status indicators were key risk determinants for malaria parasitaemia. Study findings showed a higher prevalence of asymptomatic parasitaemia in children aged 5–15 years as well as in individuals aged over 16 years compared to children aged below five years. In addition, improving HH socio-economic status and having house structural features that limit indoor malaria transmission could reduce the risk of parasitaemia and hence transmission within the community. For this area, despite high coverage of IRS and LLIN distribution, current determinants of continued malaria transmission risk remain unknown, including, but not limited to, which are the foci of transmission, whether malaria transmission occurs primarily indoors or outdoors or both, and which factors are responsible for the higher risks in males and older age groups. Evaluation of spatial covariates to explain possible malaria parasitaemia clustering, a characterization of entomological risk determinants of individual and HH malaria parasitaemia risk and identification of cost-effective measures to improve house structure features and HH socio-economic status are needed to sustainably reduce malaria transmission in Ruhuha sector.

## References

[1] Steketee RW, Sipilanyambe N, Chimumbwa J, Banda JJ, Mohamed A, Miller J (2008). National malaria control and scaling up for impact: the Zambia experience through 2006. Am J Trop Med Hyg.

[2] Karema C, Aregawi MW, Rukundo A, Kabayiza A, Mulindahabi M, Fall IS (2012). Trends in malaria cases, hospital admissions and deaths following scale-up of anti-malarial interventions, 2000–2010 Rwanda. Malar J.

[3] President’s Malaria Initiative. Rwanda Malaria Operational Plan FY 2014. Accessed 15^th^ August 2014. Available at: http://www.pmi.gov/docs/default-source/default-document-library/malaria-operational-plans/fy14/rwanda_mop_fy14.pdf?sfvrsn=8.

[4] Bousema T, Griffin JT, Sauerwein RW, Smith DL, Churcher TS, Takken W (2012). Hitting hotspots: spatial targeting of malaria for control and elimination. PLoS Med.

[5] WHO (2011). World malaria report 2011.

[6] Laishram DD, Sutton PL, Nanda N, Sharma VL, Sobti RC, Carlton JM (2012). The complexities of malaria disease manifestations with a focus on asymptomatic malaria. Malar J.

[7] Rulisa S, Kateera F, Bizimana JP, Agaba S, Dukuzumuremyi J, Baas L (2013). Malaria prevalence, spatial clustering and risk factors in a low endemic area of Eastern Rwanda: a cross sectional study. PLoS One.

[8] USAID. Demographic Health Survey Overview*.* Available at: http://www.dhsprogram.com/What-We-Do/Survey-Types/DHS.cfm. Accessed 12th May 2014.

[9] GMP/WHO. From malaria control to malaria elimination: a manual for elimination scenario planning. Available at: http://apps.who.int/iris/bitstream/10665/112485/1/9789241507028_eng.pdf. Accessed 13 March 2014.

[10] Raja A, Tridane A, Gaffar A, Lindquist T, Pribadi K (2014). Android and ODK based data collection framework to aid in epidemiological analysis. Online Journal of Public Health Informatics.

[11] Vyas S, Kumaranayake L (2006). Constructing socio-economic status indices: how to use principal components analysis. Health Policy Plan.

[12] Filmer D, Pritchett LH (2001). Estimating wealth effect without expenditure data or tears: an application to educational enrollments in states of India. Demography.

[13] WHO (2006). Systems for the early detection of malaria epidemics in Africa: an analysis of current practices and future priorities.

[14] Githinji S, Herbst S, Kistemann T, Noor AM (2010). Mosquito nets in a rural area of Western Kenya: ownership, use and quality. Malar J.

[15] Winskill P, Rowland M, Mtove G, Malima RC, Kirby MJ (2011). Malaria risk factors in north-east Tanzania. Malar J.

[16] Ghebreyesus TA, Haile M, Witten KH, Getachew A, Yohannes M, Lindsay SW (2000). Household risk factors for malaria among children in the Ethiopian highlands. Trans R Soc Trop Med Hyg.

[17] Giha HA, Rosthoj S, Dodoo D, Hviid L, Satti GM, Scheike T (2000). The epidemiology of febrile malaria episodes in an area of unstable and seasonal transmission. Trans R Soc Trop Med Hyg.

[18] Brooker S, Clarke S, Njagi JK, Polack S, Mugo B, Estambale B (2004). Spatial clustering of malaria and associated risk factors during an epidemic in a highland area of western Kenya. Trop Med Int Health.

[19] Smith T, Beck HP, Kitua A, Mwankusye S, Felger I, Fraser-Hurt N (1999). Age dependence of the multiplicity of *Plasmodium falciparum* infections and of other malariological indices in an area of high endemicity. Trans R Soc Trop Med Hyg.

[20] Smith T, Hii JL, Genton B, Muller I, Booth M, Gibson N (2001). Associations of peak shifts in age-prevalence for human malarias with bednet coverage. Trans R Soc Trop Med Hyg.

[21] Willis S, Akhwale JKL, Kaneko A, Eto H, Obonyo C, Björkman A (2004). Anemia and malaria at different altitudes in the western highlands of Kenya. Acta Trop.

[22] Sintasath D, Ghebremeskel T, Lynch M, Kleinau E, Bretas G, Shililu J (2005). Malaria prevalence and associated risk factors in Eritrea. Am J Trop Med Hyg.

[23] Beier JC, Killeen GF, Githure JI (1999). Entomologic inoculation rates and *Plasmodium falciparum* malaria prevalence in Africa. Am J Trop Med Hyg.

[24] Peterson I, Borrell LN, El-Sadr W, Teklehaimanot A (2009). Individual and household level factors associated with malaria incidence in a highland region of Ethiopia: a multilevel analysis. Am J Trop Med Hyg.

[25] Lengeler C (2004). Insecticide-treated bed nets and curtains for preventing malaria. Cochrane Database Syst Rev.

[26] Molineaux L, Gramiccia G (1980). The Garki project.

[27] Lwetoijera DW, Kiware SS, Mageni ZD, Dongus S, Harris C, Devine GJ (2013). A need for better housing to further reduce indoor malaria transmission in areas with high bed net coverage. Parasit Vectors.

[28] Takken W, Knols B (1999). Odor-mediated behavior of Afrotropical malaria mosquitoes. Annu Rev Entomol.

[29] Port GR, Boreham PFL, Bryan JH (1980). The relationship of host size to feeding by mosquitoes of the *Anopheles gambiae* Giles complex (Diptera: Culicidae). Bull Entomol Res.

[30] Guthmann JP, Hall AJ, Jaffar S, Palacios A, Lines J, Llanos-Cuentas A (2001). Environmental risk factors for clinical malaria: a case–control study in the Grau region of Peru. Trans R Soc Trop Med Hyg.

[31] Butraporn P, Sornmani S, Hungsapruek T (1986). Social, behavioural, housing factors and their interactive effects associated with malaria occurrence in east Thailand. Southeast Asian J Trop Med Public Health.

[32] West PA, Protopopoff N, Rowland M, Cumming E, Cumming E, Rand A (2013). Malaria risk factors in North West Tanzania: the effect of spraying, nets and wealth. PLoS One.

[33] Koram KA, Bennett S, Adiamah JH, Greenwood BM (1995). Socio-economic risk factors for malaria in a peri-urban area of The Gambia. Trans R Soc Trop Med Hyg.

[34] Tshikuka JG, Scott ME, Gray-Donald K, Kalumba ON (1996). Multiple infection with Plasmodium and helminths in commu- nities of low and relatively high socio-economic status. Ann Trop Med Parasitol.

[35] Ayele DG, Zewotir TT, Mwambi HG (2012). Prevalence and risk factors of malaria in Ethiopia. Malar J.

[36] Messina JP, Taylor SM, Meshnick SR, Linke AM, Tshefu AK, Atua B (2011). Population, behavioural and environmental drivers of malaria prevalence in the Democratic Republic of Congo. Malar J.

[37] Atieli H, Menya D, Githeko A, Scott T (2009). House design modifications reduce indoor resting malaria vector densities in rice irrigation scheme area in western Kenya. Malar J.

[38] Somi M, Butler J, Vahid F, Njau J, Kachur SP, Abdulla S (2007). Is there evidence for dual causation between malaria and socioeconomic status? Findings from rural Tanzania. Am J Trop Med Hyg.

[39] Clarke SE, Bogh C, Brown RC, Pinder M, Walraven GE, Lindsay SW (2001). Do untreated bednets protect against malaria?. Trans R Soc Trop Med Hyg.

